# Effect of dexmedetomidine on liver transplantation: a meta-analysis

**DOI:** 10.3389/fphar.2023.1188011

**Published:** 2023-05-22

**Authors:** Degong Jia, Shanshan Guo, Xinyi Wu, Minjie Zhao, Jiefu Luo, Mingxiang Cheng, Yajun Qin

**Affiliations:** ^1^ Department of Hepatobiliary Surgery, The Second Affiliated Hospital of Chongqing Medical University, Chongqing, China; ^2^ Department of Nephrology, The First Affiliated Hospital of Anhui Medical University, Hefei, China

**Keywords:** liver transplantation, dexmedetomedine, liver function, complication, meta-analysis

## Abstract

**Background:** Dexmedetomidine (DEX), an adjuvant anesthetic, may improve the clinical outcomes of liver transplantation (LT).

**Methods:** We summarized the relevant clinical trials of DEX in patients undergoing LT. As of 30 January 2023, we searched The Cochrane Library, MEDLINE, EMBASE, Clinical Trial.gov and the WHO ICTRP. The main outcomes were postoperative liver and renal function. The random effect model or fixed effect model was used to summarize the outcomes across centers based on the differences in heterogeneity.

**Results:** The meta-analysis included nine studies in total. Compared with the control group, the DEX group had a reduced warm ischemia time (MD-4.39; 95% CI-6.74−‐2.05), improved postoperative liver (peak aspartate transferase: MD-75.77, 95% CI-112.81−‐38.73; peak alanine transferase: MD-133.51, 95% CI-235.57−‐31.45) and renal function (peak creatinine: MD-8.35, 95% CI-14.89−‐1.80), and a reduced risk of moderate-to-extreme liver ischemia-reperfusion injury (OR 0.28, 95% CI 0.14-0.60). Finally, the hospital stay of these patients was decreased (MD-2.28, 95% CI-4.00−‐0.56). Subgroup analysis of prospective studies showed that DEX may have better efficacy in living donors and adult recipients.

**Conclusion:** DEX can improve short-term clinical outcomes and shorten the hospital stay of patients. However, the long-term efficacy of DEX and its interfering factors deserves further study.

**Systematic Review:** identifier CRD42022351664.

## Graphical Abstract

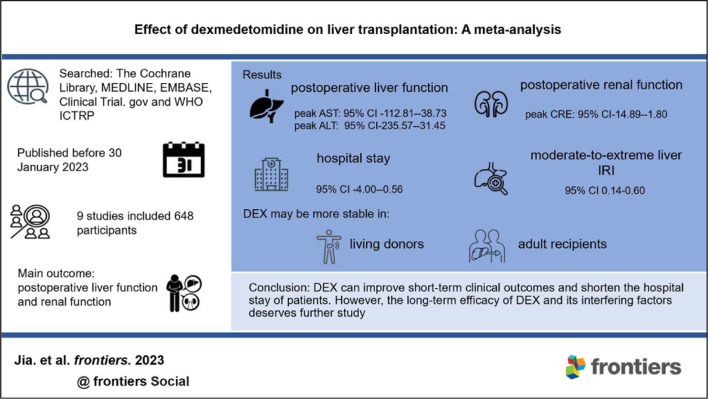

## 1 Introduction

Solid organ transplantation is currently a recognized treatment for end-stage organ disease. Due to the particularity of this operation, ischemia-reperfusion injury (IRI) is an inevitable problem (Yamada et al., 2020). In the process of liver transplantation (LT), IRI can cause graft dysfunction and vascular and biliary complications and ultimately lead to multiple organ injury (Peralta et al., 2013; Olivo et al., 2018). At the same time, inflammation and damage to the microvascular system caused by liver IRI are important factors affecting the short-term and long-term prognosis of LT (Ali et al., 2015). Therefore, effective prevention and treatment of transplanted liver IRI is crucial to the success of LT.

Perioperative hemodynamic stability during LT has a positive impact on graft function and patient recovery (Sharma et al., 2022). Dexmedetomidine (DEX) is a high-efficiency and high-selectivity alpha-2 adrenaline receptor agonist. As an auxiliary drug for general anesthesia, DEX has good perioperative hemodynamic stability and an intraoperative anesthesia retention effect. It was approved for sedation and analgesia in intensive care unit (ICU) patients in the United States in 1999 (Damian et al., 2020). Preclinical studies have shown that DEX can improve IRI in various tissues, including the liver, thus providing organ protection (Tang et al., 2020; Yu et al., 2020; Liu et al., 2022; Tao et al., 2022). However, its effectiveness and safety in IRI of transplanted livers still lack definite conclusions.

To further study and clarify the role of this convenient and economic intervention in LT, we conducted this meta-analysis. In addition, we evaluated the factors affecting the efficacy of DEX. This may provide a more effective and accurate treatment strategy for IRI of transplanted liver.

## 2 Materials and methods

This study is a summary and analysis of the clinical outcomes of DEX in patients undergoing LT. This systematic review and meta-analysis was previously registered in the International Prospective Register of Systematic Reviews (PROSPERO [CRD42022351664]) and conducted according to the PRISMA guidelines (Moher et al., 2009).

### 2.1 Search strategy

As of 30 January 2023, we searched relevant databases such as The Cochrane Library, MEDLINE, EMBASE, and online trial registration platforms such as Clinical Trial.gov and the World Health Organization International Clinical Trials Registry Platform. The retrieval was not limited by the time of publication or any other characteristics; the results were limited to English language results. See Supplementary Table S1 for the complete retrieval strategy. A total of 168 documents were retrieved, and the results were imported into Endnote X9 for further screening. Since this study only used published clinical data and did not study new human subjects, no application was submitted to IRB.

### 2.2 Selection criteria

We used Endnote X9 to remove 49 duplicate documents. Next, two independent reviewers (D.J. and S.G.) screened the title/abstract and full text of the remaining articles and recorded the number and reasons for their removal in the screening stage. The references of the included studies and related reviews were manually identified to find potential qualified tests. Any conflict between the two reviewers was resolved through discussion or the participation of a third reviewer (Y.Q.).

Before literature retrieval, we formulated the inclusion/exclusion criteria. We included original studies related to LT. The study needs to take DEX as the intervention measure, and placebo or non-DEX as the control. The related original studies about multiple organ transplantation, retransplantation and not containing the clinical data required for this study were excluded.

### 2.3 Research outcomes

The outcomes were predefined before the start of the meta-analysis and adjusted according to the actual reported data in the included studies. The main outcomes were postoperative liver function and renal function. The secondary outcomes were graft ischemia time (min), duration of surgery (h), ICU stay (d), hospital stay (d), and the occurrence of moderate-to-extreme hepatic IRI and postreperfusion syndrome (PRS). Postoperative liver function is expressed by peak aspartate transferase (AST) and alanine transferase (ALT) within 7 days after the operation, and postoperative renal function is expressed by peak blood urea nitrogen (BUN) and creatinine (CRE). Hepatic ischemia reperfusion injury (HIRI) severity was evaluated by the Rahman standard (Rahman et al., 2017).

### 2.4 Data extraction and quality assessment

The standardized form was used by two reviewers independently to extract the data included in the study, and the differences were resolved through discussion or the participation of the third reviewer. The continuous variables of each study are summarized as the mean and SD, and the dichotomous variables are summarized as the number of positive and total events. If the mean and SD of continuous variables were not reported, they were estimated based on the sample size, median, range or quartile range by using the formula proposed by Wan et al. (2014). When necessary, we contacted the original author to try to solve the ambiguity of the report data.

The quality evaluation of the randomized controlled trial was conducted independently by two authors using the Cochrane risk assessment tool (Cumpston et al., 2019); the quality evaluation of the retrospective cohort study was conducted using the Newcastle-Ottawa Quality Assessment scale.

### 2.5 Data synthesis and analysis

The data synthesis and analysis were conducted by Review Manager version 5.4, and the results report follows PRISMA guidelines. Dichotomous variables are expressed by OR values and 95% CIs; continuous variables are represented by MDs and 95% CIs, and the analysis results are visualized by forest plots. To avoid the interference of multiple comparisons on DEX efficacy judgment, we adjusted the traditional *p*-value. We obtained the multiple adjusted *p* values although 0.05 divided by the mean of 1 (no adjustment) and the number of main outcomes (Bonferroni adjustment). That is, *p* = 0.05 was used for a main outcome, and *p* = 0.033 was used for two main outcomes. Therefore, when summarizing the main outcomes, we believe that when the *p*-value is 0.033 (calculated by dividing 0.05 by [(2 + 1)/2]) or less, it is statistically significant. For the secondary outcomes, we believe that when the *p*-value is 0.014 (calculated by dividing 0.05 by [(6 + 1)/2]) or less, it is statistically significant (Jakobsen et al., 2014; Jakobsen et al., 2016). Two in the above equation represents two main outcomes (postoperative liver function and renal function); six means six secondary outcomes (graft ischemia time, duration of surgery, ICU stay, hospital stay, and the occurrence of moderate-to-extreme hepatic IRI and PRS).

The statistical heterogeneity among the studies was evaluated by the chi2 and I2 tests. Heterogeneity was considered when P Heterogeneity< 0.1 or I2 >50%. When statistical heterogeneity existed among the studies, the random effect model was used to summarize the data; otherwise, the fixed effect model was used (Jia et al., 2023). At the same time, we plan to use subgroup analysis to find the influencing factors of DEX efficacy.

### 2.6 Subgroup analysis

This study is the first to explore the factors affecting the efficacy of DEX. It is planned to conduct hierarchical analysis based on different countries (developed or non-developed), research types (retrospective or prospective), donor types (deceased donor or living donor) and different populations (adult or pediatric) to find potential sources of differences and the best clinical environment for DEX in LT. This study also aimed to identify the applicable population for DEX to avoid wasting medical resources.

## 3 Results

### 3.1 Summary of included studies

**FIGURE 1:**
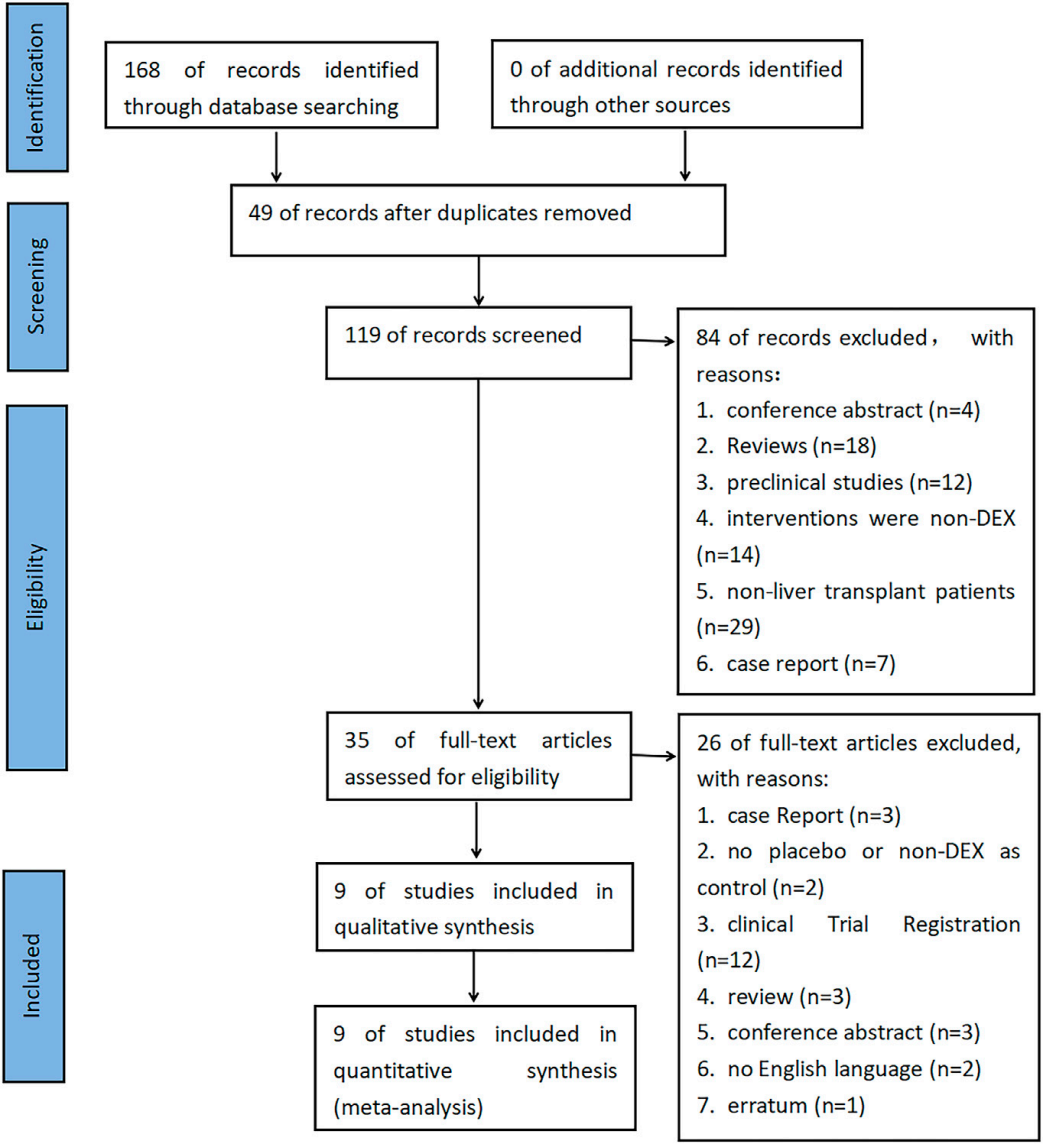
PRISMA flow chart of the literature retrieval and screening process.

A total of 168 documents were retrieved from the database, and 49 duplicate documents were removed. The title/abstract and full text of the remaining 119 documents were reviewed. Finally, 110 documents were excluded because they did not meet the preset inclusion criteria. No new clinical trial was found after searching the clinical trial registration platform ClinicalTrial.gov and WHO ICTRP. Finally, nine studies were included in the meta-analysis, with a total of 648 participants (Choi et al., 2016; Fayed et al., 2016; Sayed and Yassen, 2016; Xu et al., 2016; Lee et al., 2020; Lee et al., 2021; Ustun et al., 2021; Zhang et al., 2021; Zhang et al., 2022). See Figure 1 for the combined search results. Table 1 describes the basic characteristics of the included studies. The nine included studies were all single-center trials, including four prospective randomized controlled trials and five retrospective cohort studies. Two studies were conducted in children, and the other seven studies were conducted in adults. Table 2 is a detailed description of all expected research results. Table 3 is a detailed description of all subgroup analysis results.

**TABLE 1:** Characteristics of the included studies.

Author, year	No. of patients DEX control		Age DEX control		Male% DEX control		MELD score DEX control		Intervention	Control	Study type
Fayed NA. 2016	20	20	50.6	52.47	85	90	15.7	16.1	a continuous intraoperative infusion of 0.8 l g/kg/h of DEX; infusion was started after the induction of anesthesia and continued until the end of surgery	placebo	RCT
J.Y. Choi. 2016	16	26	53.83	49.45	81.2	84.6	22	23.26	DEX was administrated at a continuous intravenous infusion rate of 0.3 mg/kg/h without a loading dose	haloperidol was administrated as an intravenous bolus or intramuscular injection of 3 mg–5 mg	retrospective cohort study
Lee, C. F. 2021	23	26	53.0	58.5	69.6	57.7	14	16	administered DEX through continuous infusion (0.2 mcg/kg/h) to the recipients upon arrival to the ICU after operation. The maximum infusion time was 24 h	without the use of DEX when undergoing LT.	retrospective cohort study
Lee, H. 2020	100	101	56.35	55.70	71.0	72.3	11.70	12.35	DEX (0.1 mcg/kg/h) was administered for patients during anesthesia and through postoperative day 2	0.9% saline (0.1 mcg/kg/h) was administered for patients during anesthesia and through postoperative day 2	RCT
Sayed, E. 2016	20	20	43.3	44.8	70	80	14.3	14.2	a continuous infusion of DEX starting at 0.5 mic/kg/h (0.2–0.7 mic/kg/h); started with the induction of anesthesia and continued until the end of skin wound closure	saline boluses and placebo infusions	RCT
Ustun, Y. B. 2021	11	10	49.01	51.17	82	60	17.40	19.58	DEX infusions were titrated between 0.2 and 0.7 mcg/kg/h and 0.03 and 0.05 mg/kg/h, respectively, to maintain a stable cardiopulmonary response and continued for 12 h in the postoperative ICU.	Midazolam infusions were titrated between 0.2 and 0.7 mcg/kg/h and 0.03 and 0.05 mg/kg/h, respectively, to maintain a stable cardiopulmonary response and continued for 12 h in the postoperative ICU.	retrospective cohort study
Xu, G. 2016	40	40	51.03	48.83	50	55	16.60	18.13	an initial dose of DEX at 1 μg/kg for 10 min followed by a continuous infusion at 0.3 μg/kg/h until the end of surgery	a saline treatment	RCT
Zhang, L. 2021	28	26	3.57	2.82	71.4	53.8	N/S	N/S	DEX at 0.4 μg/kg/h from incision to the end of the operation	Nothing	retrospective cohort study
Zhang, L. 2022	62	59	1.87	1.94	50	49.2	N/S	N/S	a continuous infusion of DEX at 0.4 g/kg/h without a loading dose from incision to the end of surgery	Nothing	retrospective cohort study

Abbreviations: DEX: dexmedetomidine; N/S: not stated; RCT: randomized controlled trial; ICU: intensive care unit.

**TABLE 2:** Summary of meta-analysis results of DEX in LT.

Outcomes	No. of included studies	Total number of participants in the DEX and control groups	Heterogeneity	Effect estimation	*p*-value
Peak AST (U/L)	3	110 in the DEX group and 105 in the control group	I^2^ 0%	MD -75.77 95% CI-112.81−‐38.73	<0.0001
Peak ALT (U/L)	3	110 in DEX and 105 in control	I^2^ 60%	MD -133.51 95% CI-235.57−‐31.45	0.01
Peak BUN (mmol/L)	2	90 in the DEX group and 85 in the control group	I^2^ 89%	MD 0.06 95% CI-2.71–2.82	0.97
Peak CRE (µmol/L)	3	110 in the DEX group and 105 in the control group	I^2^ 67%	MD -8.35 95% CI-14.89−‐1.80	0.01
Warm ischemia time (min)	3	110 in the DEX group and 105 in the control group	I^2^ 19%	MD -4.39 95% CI-6.74−‐2.05	0.0002
Cold ischemia time (min)	4	121 in the DEX group and 115 in the control group	I^2^ 7%	MD -7.08 95% CI-15.95–1.78	0.12
Hospital stay d)	4	230 in the DEX group and 226 in the control group	I^2^ 35%	MD -2.28 95% CI-4.00−‐0.56	0.01
ICU stay d)	7	289 in the DEX group and 298 in the control group	I^2^ 48%	MD -0.05 95% CI-0.24–0.14	0.60
Duration of surgery h)	4	180 in the DEX group and 181 in the control group	I^2^ 0%	MD 0.09 95% CI-0.28–0.45	0.64
moderate-to-extreme HIRI	2	90 in the DEX group and 85 in the control group	I^2^ 0%	OR 0.28 95% CI 0.14–0.60	0.001
Postreperfusion syndrome	3	110 in the DEX group and 105 in the control group	I^2^ 68%	OR 0.72 95% CI 0.19–2.74	0.63

Abbreviations: DEX: dexmedetomidine; AST: aspartate transferase; ALT: alanine transferase; BUN: blood urea nitrogen; CRE: creatinine; ICU: intensive care unit; HIRI: hepatic ischemia reperfusion injury.

**TABLE 3:** Summary of the effects of different factors on the efficacy of DEX.

Outcome	Subgroup	No. of studies	Population size	Effect estimation (95% CI)	I^2^ statistic (%)
Peak AST (U/L)	Retrospective	2	175	-264.09-18.52	0
	Prospective	1	40	-110.68−‐33.92	-
	Non-developed	3	215	-112.81−‐38.73	0
	Deceased donor	1	54	-1,275.23-486.77	-
	Living donor	2	161	-112.28−‐38.13	0
	Adult	1	40	-110.68−‐33.92	-
	Pediatric	2	175	-264.09-18.52	0
Peak ALT (U/L)	Retrospective	2	175	-508.38-24.44	60
	Prospective	1	40	-114.30−‐56.10	-
	Non-developed	3	215	-235.57−‐31.45	60
	Deceased donor	1	54	-770.09−‐93.69	-
	Living donor	2	161	-122.37−‐57.94	4
	Adult	1	40	-114.30−‐56.10	-
	Pediatric	2	175	-508.38-24.44	60
Peak BUN (mmol/L)	Retrospective	2	175	-2.71-2.82	89
	Non-developed	2	175	-2.71-2.82	89
	Deceased donor	1	54	-0.10-3.26	-
	Living donor	1	121	-1.94−‐0.56	-
	Pediatric	2	175	-2.71-2.82	89
Peak CRE (µmol/L)	Retrospective	2	175	-9.12−‐1.14	0
	Prospective	1	40	-25.14−‐8.45	-
	Non-developed	3	215	-14.89−‐1.80	67
	Deceased donor	1	54	-11.39-1.15	-
	Living donor	2	161	-21.87-0.89	82
	Adult	1	40	-25.14−-8.45	-
	Pediatric	2	175	-9.12−‐1.14	0
Warm ischemia time (min)	Retrospective	2	175	-10.58−‐0.54	58
	Prospective	1	40	-11.20-4.20	-
	Non-developed	3	215	-6.74−‐2.05	19
	Deceased donor	1	54	-15.22−‐2.78	-
	Living donor	2	161	-6.16−‐1.11	0
	Adult	1	40	-11.20-4.20	-
	Pediatric	2	175	-10.58−‐0.54	58
Cold ischemia time (min)	Retrospective	3	196	-19.77−‐0.18	0
	Prospective	1	40	-14.83-26.83	-
	Non-developed	4	236	-15.95-1.78	7
	Deceased donor	1	54	-76.67-46.67	-
	Living donor	2	161	-16.25-1.71	48
	Adult	2	61	-13.03-28.11	0
	Pediatric	2	175	-20.24−‐0.59	0
Hospital stay (d)	Retrospective	2	175	-10.21−‐1.16	0
	Prospective	2	281	-3.57-0.16	24
	Developed	1	201	-3.28-2.34	-
	Non-developed	3	255	-5.55−‐1.19	3
	Deceased donor	1	54	-9.98-2.26	-
	Living donor	2	322	-10.68-3.63	75
	Adult	2	281	-3.57-0.16	24
	Pediatric	2	175	-10.21−‐1.16	0
ICU stay (d)	Retrospective	4	266	-0.58-0.22	64
	Prospective	3	321	-0.23-0.20	26
	Developed	2	243	-0.18-0.30	79
	Non-developed	5	344	-0.55-0.08	13
	Deceased donor	1	54	-0.85-0.65	-
	Living donor	4	411	-0.21-0.20	50
	Adult	5	412	-1.03-0.16	65
	Pediatric	2	175	-0.43-0.42	0
Duration of surgery (h)	Prospective	4	361	-0.28-0.45	0
	Developed	1	201	-0.51-0.37	-
	Non-developed	3	160	-0.22-1.09	0
	Living donor	3	281	-0.37-0.44	0
	Adult	4	361	-0.28-0.45	0
Moderate-to-Extreme HIRI	Retrospective	2	175	0.14-0.60	0
	Non-developed	2	175	0.14-0.60	0
	Deceased donor	1	54	0.05-0.84	-
	Living donor	1	121	0.14-0.79	-
	Pediatric	2	175	0.14-0.60	0
Post-reperfusion syndrome	Retrospective	2	175	0.07-6.50	84
	Prospective	1	40	0.19-3.13	-
	Non-developed	3	215	0.19-2.74	68
	Deceased donor	1	54	0.05-0.84	-
	Living donor	2	161	0.54-3.50	9
	Adult	1	40	0.19-3.13	-
	Pediatric	2	175	0.07-6.50	84
	Intraoperative	3	215	0.19-2.74	68

Abbreviations: DEX: dexmedetomidine; AST: aspartate transferase; ALT: alanine transferase; BUN: blood urea nitrogen; CRE: creatinine; ICU: intensive care unit; HIRI: hepatic ischemia reperfusion injury.

**FIGURE 2:**
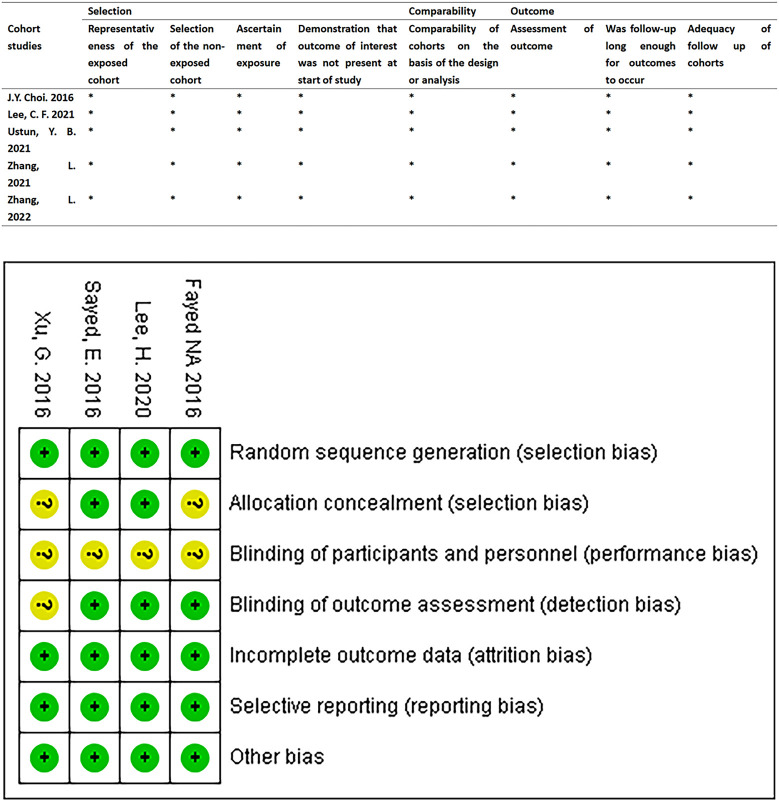
Quality assessment results of the included studies. RCTs were assessed using the Cochrane Risk of Bias Assessment Tool. Retrospective cohort studies were assessed using the Newcastle-Ottawa Quality Assessment scale.

We found the risk of ambiguous bias in some studies because the author did not describe any relevant details. Figure 2 describes the quality evaluation results of the included studies. The overall bias of each study was low. The quality of all the included studies was generally good, and none of them showed poor design.

### 3.2 Primary outcome

#### 3.2.1 Postoperative liver function

**FIGURE 3:**
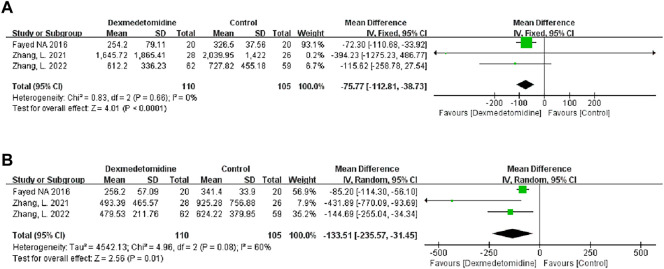
Forest plots of the effect of DEX on postoperative peak aspartate transferase **(A)** and alanine transferase **(B)**.

We used the peak AST/ALT within 7 days after transplantation to reflect postoperative liver function. Three studies recorded the level of peak AST/ALT after LT, and 110 participants received DEX. Meta-analysis showed that compared with the control group, the group with perioperative DEX infusion had significantly reduced peak AST (MD -75.77; 95% CI-112.81−‐38.73) and ALT levels (MD -133.51; 95% CI-235.57−‐31.45) (Figure 3) and improved postoperative liver function. Subgroup analysis of prospective studies showed that the results were stable in living donors and adult recipients (Table 3).

#### 3.2.2 Postoperative renal function

**FIGURE 4:**
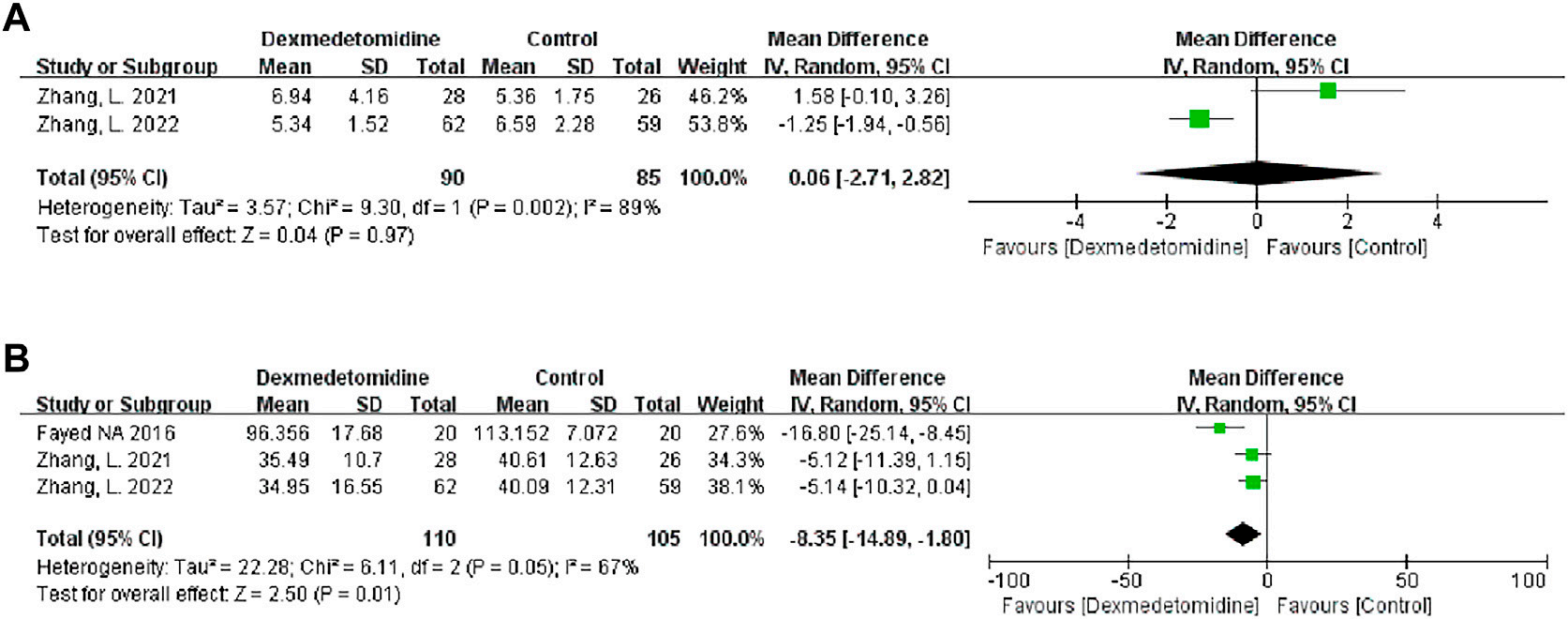
Forest plots of the effect of DEX on postoperative peak blood urea nitrogen **(A)** and creatinine **(B)**.

We used the peak BUN and CRE levels within 7 days after transplantation to reflect postoperative renal function. Two studies described the level of peak BUN after LT, and 90 participants received DEX. Meta-analysis showed that the infusion of DEX did not significantly improve the peak BUN (MD 0.06; 95% CI-2.71-2.82) level (Figure 4A). Three studies described the level of peak CRE after surgery, and 110 people received DEX. Meta-analysis showed that the DEX group had significantly reduced peak CRE levels (MD -8.35; 95% CI-14.89−‐1.80) (Figure 4B) after the operation compared with the control group. Subgroup analysis showed that donor type could affect the role of DEX in postoperative renal function (Table 3).

### 3.3 Secondary outcome

#### 3.3.1 Graft ischemia time

**FIGURE 5:**
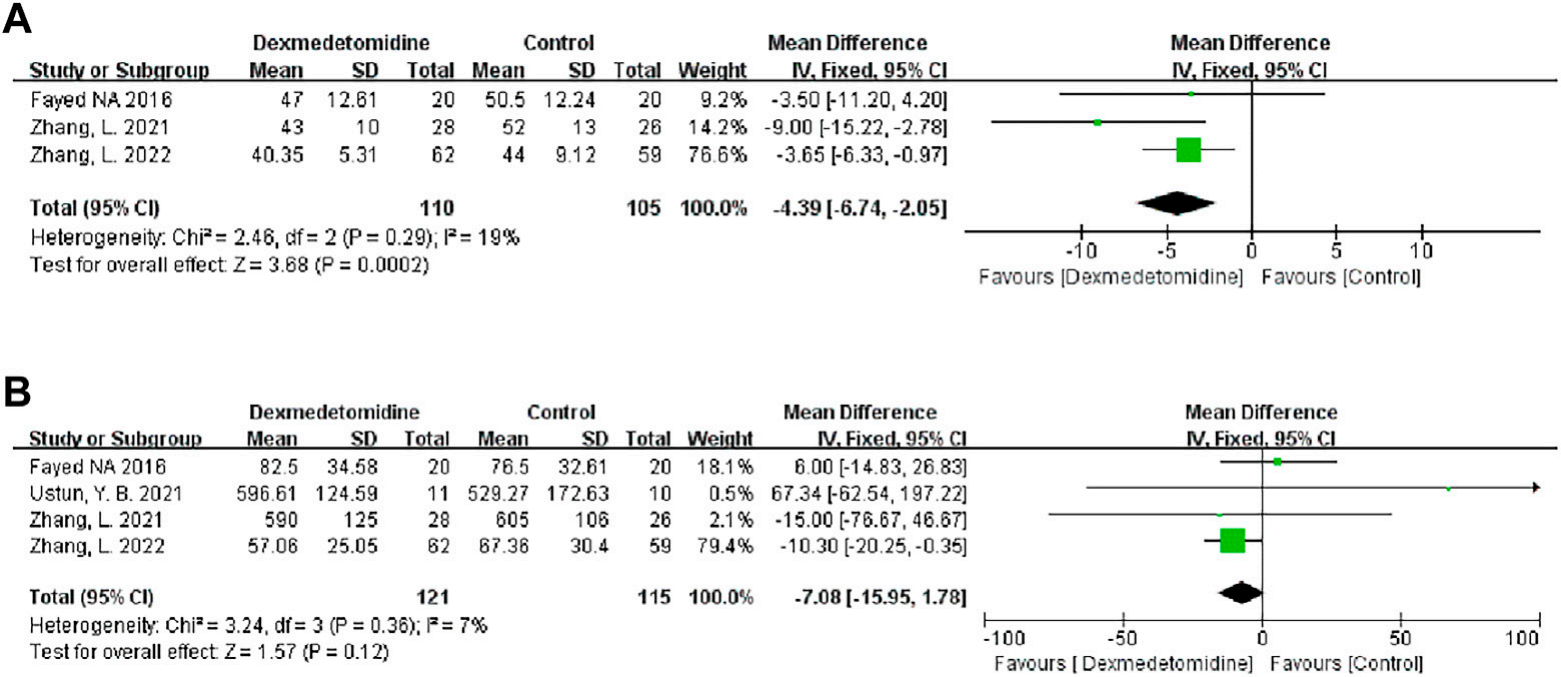
Forest plots of the effect of DEX on warm ischemia time **(A)** and cold ischemia time **(B)**.

We analyzed the effect of DEX on warm ischemia time (WIT) and cold ischemia time (CIT). Three studies recorded WIT during the operation, and 110 people received DEX. Meta-analysis showed that the DEX group had a significantly shortened WIT compared with the control group (MD -4.39; 95% CI-6.74−‐2.05) (Figure 5A). Subgroup analysis showed that the study type had an impact on this result, and it was more stable among child recipients (Table 3).

Four studies recorded CIT, and 121 participants received DEX. Meta-analysis did not show any effect of DEX on the CIT (MD -7.08; 95% CI-15.95-1.78) (Figure 5B). Subgroup analysis showed that the study type and age of the recipients had a significant impact on this result (Table 3).

#### 3.3.2 Duration of surgery

**FIGURE 6:**
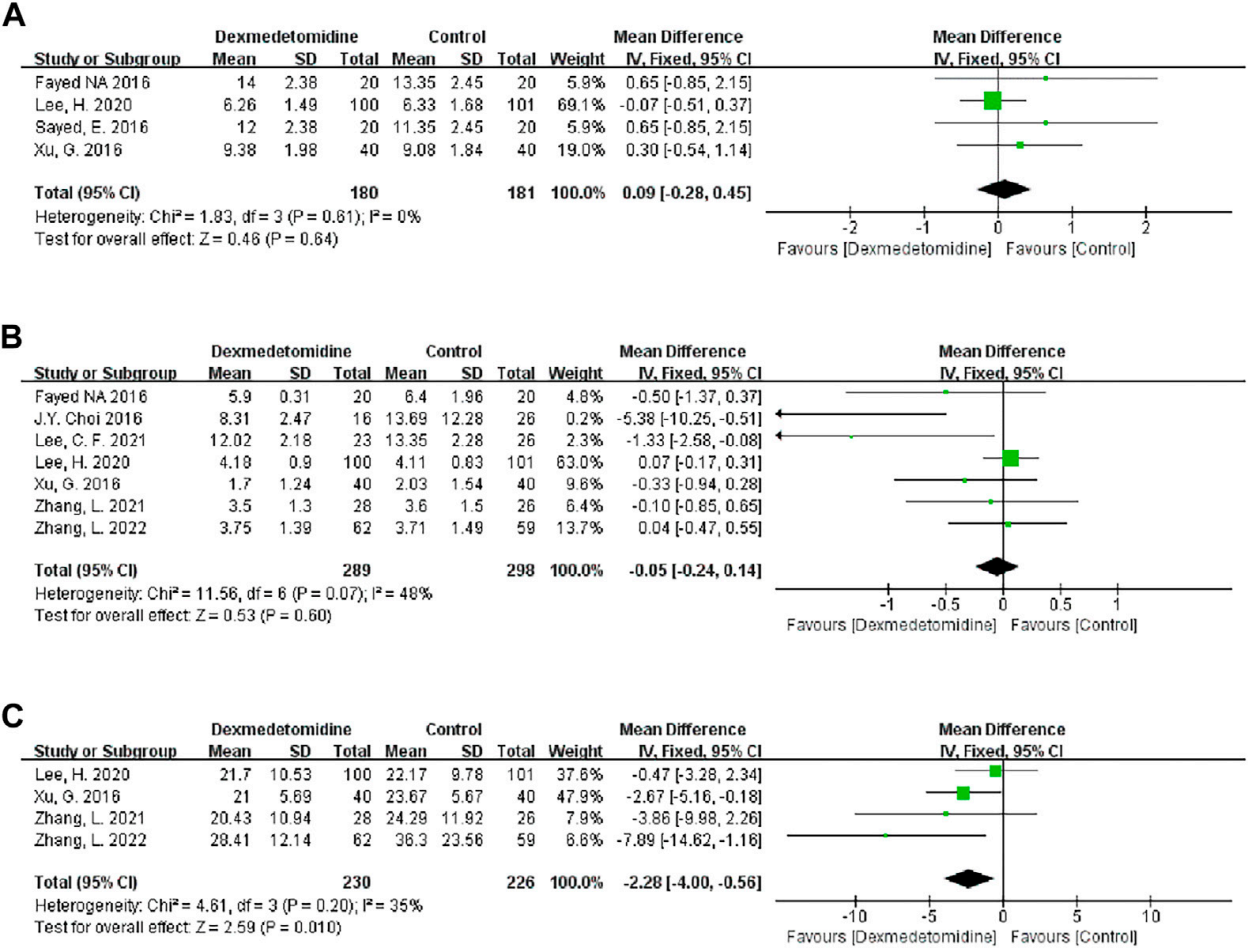
Forest plots of the effect of DEX on the duration of surgery **(A)**, ICU stay **(B)** and hospital stay **(C)**.

Four studies recorded the duration of surgery, and 180 people received DEX. Meta-analysis showed that DEX did not change the operation duration (MD 0.09; 95% CI-0.28-0.45) (Figure 6A). Subgroup analysis showed that the study type, country type and recipient age could change the effect of DEX on the operation duration (Table 3).

#### 3.3.3 ICU stay

Seven studies described the ICU stay of recipients, and 289 people received DEX. Meta-analysis did not show that DEX had a significant effect on the ICU stay of patients (MD -0.05; 95% CI-0.24-0.14) (Figure 6B). The result was stable in all subgroups (Table 3).

#### 3.3.4 Hospital stay

Four studies described the impact of DEX on the hospital stay of patients, and 230 participants received DEX. Meta-analysis showed that DEX significantly reduced the hospital stay of patients compared with the control group (MD -2.28; 95% CI-4.00−‐0.56) (Figure 6C). Subgroup analysis of the retrospective study showed that this result was stable in non-developed countries and child recipients (Table 3).

#### 3.3.5 Moderate-to-extreme hepatic IRI

**FIGURE 7:**
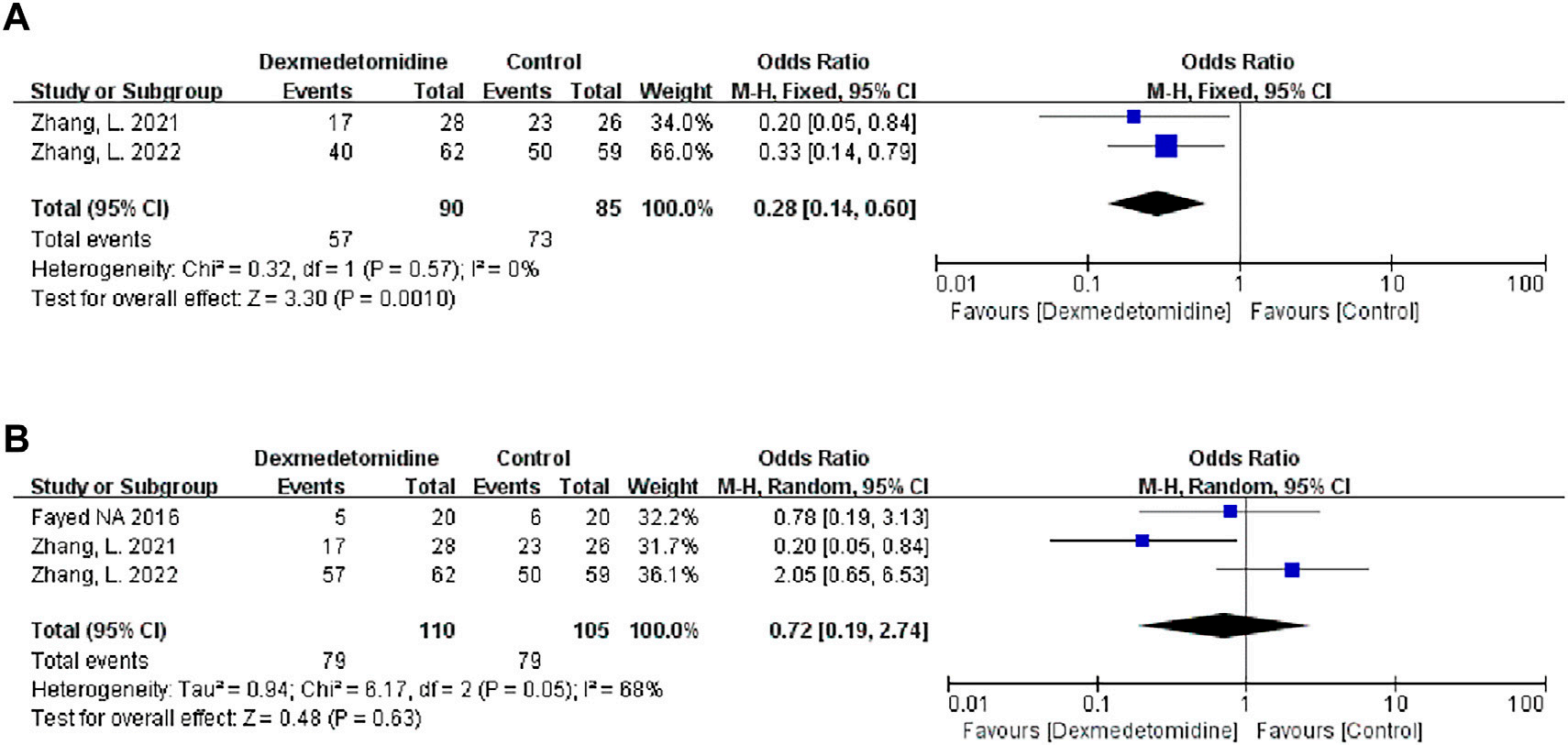
Forest plots of the effect of DEX on moderate-to-extreme hepatic IRI **(A)** and postreperfusion syndrome **(B)**.

Two studies described the occurrence of moderate-to-extreme liver IRI, of which 90 participants received DEX. Meta-analysis showed that the DEX group had a significantly reduced incidence of moderate-to-extreme hepatic IRI compared with the control group (OR 0.28; 95% CI 0.14-0.60) (Figure 7A). The result remained stable in all subgroups (Table 3).

#### 3.3.6 Postreperfusion syndrome

Three studies described the occurrence of PRS, and 110 people received DEX treatment. Meta-analysis showed that DEX did not affect the occurrence of PRS (OR 0.72; 95% CI 0.19-2.74) (Figure 7B). Subgroup analysis showed that donor type had an impact on this result (Table 3).

## 4 Discussion

This meta-analysis is a comprehensive analysis of the clinical prognosis data of nine studies on DEX treatment of LT, involving 648 participants from South Korea, China and other countries. Encouragingly, the infusion of DEX significantly reduced the levels of peak AST/ALT and CRE in the early postoperative period. DEX can significantly reduce WIT and the incidence of moderate-to-extreme hepatic IRI and ultimately significantly reduce the length of hospital stay of patients. In general, DEX plays an active role in LT, which is more stable in living donors, prospective studies and adult recipients. However, considering the small number of participants in this study, the above results still need to be interpreted with caution. Further prospective, double-blind, multicenter studies are essential to confirm the efficacy of DEX in LT and its influencing factors.

DEX can alleviate early postoperative liver function, which is reflected in DEX significantly reducing the level of peak AST/ALT. On the one hand, this effect may be because DEX can reduce the level of the leukocyte adhesion molecule ICAM-1 and the migration of leukocytes to the inflammatory region, which reduces the damage to liver endothelial cells, and DEX also inhibits the activation of the intrinsic apoptotic cascade reaction, which ultimately restores liver function (Vollmar et al., 1995; Fayed et al., 2016; Hemsinli et al., 2022). On the other hand, this result is consistent with the finding that DEX reduces moderate-to-extreme hepatic IRI, which is the inducing factor of graft dysfunction. DEX has been shown to reduce hepatic IRI through multiple pathways, including decreasing oxidative stress, endoplasmic reticulum stress, and apoptotic pathways (Zhang et al., 2023). Notably, the total sample size of the current study may not be sufficient to reveal the role of DEX in postoperative liver function. Therefore, this result should be interpreted with caution.

Because of the high incidence rate of kidney injury after LT, it is necessary to explore this field. This study is the first summary of clinical evidence related to DEX in renal function after LT and shows that DEX has a certain protective effect, which is reflected in that DEX reduces the level of peak CRE. According to previous research reports, this effect may be related to DEX as an alpha-2 adrenergic receptor agonist. Alpha-2 adrenergic receptors are widely distributed in renal tubules and their surrounding vascular systems, and their activation can regulate endothelial nitric oxide synthase to induce vasodilation, thus increasing the glomerular filtration rate and urine volume and improving the damage to renal function caused by LT (Nong et al., 2016). However, our study did not find an effect of DEX on BUN, which may be because the mechanism of organ dysfunction caused by liver IRI is relatively complex, and it is difficult to play a complete protective role through a single drug or medium.

Studies have shown that shortening the WIT can reduce the risk of early graft dysfunction and graft loss at 1 and 5 years after surgery and play a protective role in the prognosis of LT (Al-Kurd et al., 2021). Meanwhile, the WIT is closely related to the surgical skills of LT and is basically considered a fixed time in the clinical environment of contemporary LT. Instead, we found that the non-mechanical intervention “drug injection” reduced the WIT during the operation. This may be due to the small number of studies and participants, and 66.67% of the included studies were retrospective studies, resulting in the deviation of the research results. In the future, more and larger prospective clinical trials are needed to confirm whether DEX can reduce the intraoperative WIT.

Furthermore, we found that DEX shortened the hospital stay of patients. The length of hospital stay is considered to be closely related to infection risk, medical care expenditure and other postoperative outcomes (Du et al., 2022). A previous study showed dexmedetomidine acted as an alpha-2 receptor agonist and sodium channel inhibitor to regulate the function of locus coerulus and dorsal horn, reducing postoperative stress response and alleviating anxiety (Weerink et al., 2017; Wiatrowski, 2021). Thereby it could accelerate postoperative recovery in transplant patients. This efficacy of DEX has many clinical benefits. On the one hand, the reduction in hospital stay can effectively reduce the incidence of complications such as nosocomial infection and the overall hospital costs of patients and ultimately reduce the economic, physical and mental burden of patients. On the other hand, the short length of hospitalization has accelerated the turnover rate of hospital beds, enabling candidates on the waiting list for LT to undergo surgery as soon as possible and promoting the efficient use of medical resources.

At the same time, we analyzed the influencing factors of DEX treatment for LT. From the results of the subgroup analysis, DEX seems more effective and stable in living donors, prospective studies, and adult recipients. Compared with deceased donors, living donors have many advantages, such as a shorter CIT and more opportunities for medical optimization before transplantation (Tran and Humar, 2021). It is not surprising that living donors have better postoperative recovery. With regard to different types of studies, the retrospective study did not develop a standard anesthesia and surgical plan before the study, and the dosage and use of DEX were not determined, so the evaluation of the efficacy of DEX was inevitably biased. Moreover, there is no unified standard for the perioperative treatment and nursing of liver transplant patients, which may lead to relatively poor results in retrospective studies. Regarding different groups of liver transplant recipients, compared with children, the intraoperative and postoperative nursing technology in adults is relatively more mature. Moreover, due to the characteristics of physical structure, it is more difficult for children to match the liver of proper size, and the formation rate of hepatic vein thrombosis after transplantation is higher (Rawal and Yazigi, 2017; Nickel et al., 2022). Therefore, the prognosis of LT in children may be relatively poor.

In addition, preclinical studies have shown that other commonly used anesthetics and anesthesia adjuncts, such as sevoflurane, isoflurane and propofol, could also alleviate IRI (Hausburg et al., 2020; Chen et al., 2022; Benoit et al., 2023). For example, sevoflurane alleviates hepatic cell death induced by IRI by reducing oxidative stress, inhibiting the formation or opening of mitochondrial permeability transition pore and NF-κB signaling pathway, and increasing the expression of hypoxia-inducing factors (Benoit et al., 2023). Therefore, sevoflurane is also a potential drug for reducing hepatic IRI. In LT, pharmacological methods with the same efficacy seem more applicable compared to invasive surgical strategies such as ischemic preconditioning (Jeong et al., 2017). However, it is uncertain whether these anesthetic drugs have any benefits in the clinical outcomes of liver transplant patients, and further prospective clinical trials are needed to further verify.

### 4.1 Strengths and limitations

To the best of our knowledge, this is the first systematic review and meta-analysis to study the role of DEX in LT. Compared with any individual study, this study followed known guidelines and standards in the review and reporting process and provided clinical evidence for DEX in LT through rigorous meta-analysis. Our results support clinicians in choosing this economic and convenient intervention method in the process of LT. We found the possible factors that affect the efficacy of DEX. Of course, this study also has certain deficiencies. First, the documents included in this study are all single-center studies. The surgical and nursing skills of the transplant center and the basic clinical characteristics of patients inevitably lead to potential differences between the studies. Second, this study lacks objective evaluation indicators of DEX in LT, such as its impact on anesthesia demand, intraoperative hemodynamic stability and blood glucose level. Third, clinical data on adverse drug reactions associated with DEX and its effects on the heart, pancreas and other organs after LT were not reported. Fourth, there were no crucial short-term and long-term follow-up data after the operation, including the description of graft survival and patient survival. Fifth, the dose-dependent effect of DEX on LT was not reported, and the optimal injection time of DEX was also not clear in this study. In addition, it is not clear whether preoperative prophylactic medication and postoperative continuous medication have better protective effects. Finally, this study contains clinical data from multiple countries and different years, and there is inevitably heterogeneity between studies. Although we tried to find the potential confounding factors that affect the efficacy of DEX, the existence of heterogeneity and the lack of understanding of the relevant mechanisms that affect the prognosis of LT mean that our research may still be biased.

## 5 Conclusion

In this study, we evaluated the efficacy of DEX on LT. In general, DEX improves the postoperative liver function and renal function of patients, reduces the postoperative hospitalization time of patients, and plays a favorable role in the prognosis of LT. Its role in living donors and adult recipients is more stable. DEX, as a low-cost intervention with few side effects, is a promising protective factor for LT. However, before DEX is widely used, more and larger clinical trials are still needed to further confirm its efficacy and side effects.

## Data Availability

The original contributions presented in the study are included in the article/Supplementary Material, further inquiries can be directed to the corresponding author.
