# Supplementary material for: Effect of dexmedetomidine on liver transplantation: a meta-analysis
Source: Front Pharmacol. 2023 May 22;14:1188011. doi: 10.3389/fphar.2023.1188011 (PMC10245273; doi:10.3389/fphar.2023.1188011)
Supplement: Supplementary file 1 [file Table1.DOCX]

Supplementary Material

Effect of dexmedetomidine on liver transplantation: A meta-analysis

Degong Jia^1,^ ^†^, Shanshan Guo^2, †^, Xinyi Wu^1^, Minjie Zhao^1^, Jiefu Luo^1^, Mingxiang Cheng^1^, Yajun Qin^1, *^

*** Correspondence:** Yajun Qin: [qinyajun@hospital.cqmu.edu.cn](mailto:qinyajun@hospital.cqmu.edu.cn)

^†^ These authors contributed equally to this work and share first authorship

# 1 Supplementary Figures and Tables

## Supplementary Tables

**Supplementary Table 1:** Search Strategy

| **Database** | **Search Strategy** |
| --- | --- |
| **The Cochrane Library** | **#1MeSH:liver transplantation**  **#2 (liver or hepatic) AND (transplant* or graft*)**  **#3 #1or#2**  **#4 MeSH descriptor: [****Dexmedetomidine]**  **#5 MeSH descriptor: [Adrenergic alpha‐Agonists]**  **#****6(Precedex or Dexmedetomidin*) or ((****adren?ergic or alpha) near agonist*)**  **#7 #4or#5or#6**  **#8 #3and#7** |
| **MEDLINE**  **(pubmed)** | **#1liver transplantation/**  **#2 (liver or hepatic) AND (transplant* or graft*)**  **#3 #1or#2**  **#4 Dexmedetomidine/**  **#5** **Adrenergic alpha‐Agonists/**  **#6 Precedex or Dexmedetomidin***  **#7 (adren?ergic or alpha) adj3 agonist***  **#8 #4or#5or#6or#7**  **#9 #3and#8** |
| **EMBASE** | **#1 liver transplantation/**  **#2 (liver or hepatic) AND (transplant* or graft*)**  **#3 #1or#2**  **#4 Dexmedetomidine/**  **#5 Adrenergic alpha‐Agonists/**  **#6 Precedex or Dexmedetomidin***  **#7 (adren?ergic or alpha) adj3 agonist***  **#8 #4or#5or#6or#7**  **#9 #3and#8** |
| **ClinicalTrial.gov** | **#1 liver transplantation**  **#2 Dexmedetomidine** |
| **WHO ICTRP** | **#1 liver transplantation**  **#2 Dexmedetomidine** |
